# Generalized Stoichiometry and Biogeochemistry for Astrobiological Applications

**DOI:** 10.1007/s11538-021-00877-5

**Published:** 2021-05-18

**Authors:** Christopher P. Kempes, Michael J. Follows, Hillary Smith, Heather Graham, Christopher H. House, Simon A. Levin

**Affiliations:** 1 The Santa Fe Institute, Santa Fe, NM USA; 2 Department of Earth, Atmospheric, and Planetary Sciences, Massachusetts Institute of Technology, Cambridge, MA USA; 3 Department of Geosciences, Pennsylvania State University, University Park, PA, USA; 4 NASA Goddard Spaceflight Center, Greenbelt, MD USA; 5 Department of Ecology and Evolutionary Biology, Princeton University, Princeton, NJ USA; 6 Catholic University of America, Washington, DC USA

## Abstract

A central need in the field of astrobiology is generalized perspectives on life that make it possible to differentiate abiotic and biotic chemical systems McKay (2008). A key component of many past and future astrobiological measurements is the elemental ratio of various samples. Classic work on Earth’s oceans has shown that life displays a striking regularity in the ratio of elements as originally characterized by Redfield (Redfield 1958; Geider and La Roche 2002; Eighty years of Redfield 2014). The body of work since the original observations has connected this ratio with basic ecological dynamics and cell physiology, while also documenting the range of elemental ratios found in a variety of environments. Several key questions remain in considering how to best apply this knowledge to astrobiological contexts: How can the observed variation of the elemental ratios be more formally systematized using basic biological physiology and ecological or environmental dynamics? How can these elemental ratios be generalized beyond the life that we have observed on our own planet? Here, we expand recently developed generalized physiological models (Kempes et al. 2012, 2016, 2017, 2019) to create a simple framework for predicting the variation of elemental ratios found in various environments. We then discuss further generalizing the physiology for astrobiological applications. Much of our theoretical treatment is designed for *in situ* measurements applicable to future planetary missions. We imagine scenarios where three measurements can be made—particle/cell sizes, particle/cell stoichiometry, and fluid or environmental stoichiometry—and develop our theory in connection with these often deployed measurements.

## Introduction

Since the recognition that life on Earth is characterized by a striking regularity in the ratio of elements, as originally characterized by Redfield (Redfield 1958; Geider and La Roche 2002; Eighty years of Redfield 2014), stoichiometric ratios have been a primary target of astrobiological measurements and theories (Elser 2003; Young et al. 2014). From an astrobiological perspective the natural questions that emerge are how much variation exists in these ratios across the range of environments and biological diversity on Earth, how different ratios could have been in time, how different they could be for non-Terran life, and how they depend on planetary composition (Elser 2003; Young et al. 2014; Anbar 2008; Chopra and Lineweaver 2008; Lineweaver and Chopra 2012; Neveu et al. 2016; Wang et al. 2018; Geider and La Roche 2002). On Earth the Redfield ratio is known to vary significantly due to both environmental and physiological effects that have been considered in ecological and biogeochemical theories (e.g. Geider and La Roche 2002; Klausmeier et al. 2004a, b, 2008; Loladze and Elser 2011; Neveu et al. 2016; Sterner et al. 2008; Vrede et al. 2004; Elser et al. 2000; Kerkhoff et al. 2005; Elser et al. 2010; Liefer et al. 2019; Finkel et al. 2016a, b). For life with a different evolutionary history we need new approaches that are able to generalize organism physiology and define when the stoichiometric ratios associated with life are distinct and distinguishable from the environment.

Our general approach here is to first focus on the macromolecules and physiology shared by all of life on Earth. For the macromolecules we are interested in components like proteins, nucleic acids, and cell membranes. For the shared physiology we consider processes such as growth rates, nutrient uptake, and nutrient storage, some of which are derivable from the macromolecular composition of cells. In thinking about the applicability of these two perspectives to life anywhere in the universe it is important to note that the specific set of macromolecules might vary significantly while the general physiological processes might be more conserved. However, our treatment of the macromolecules is easily generalized if one makes two assumptions: 1) that life elsewhere shares a set of macromolecules, even if that set is very different from Terran life, and 2) that those macromolecules fall along systematic scaling relationships. Throughout this paper we operate within these two assumptions and first address the observation and implications of (2), before moving on to a general treatment of physiological scaling which abstracts the underlying details of (1). Throughout we go back and forth between the patterns observed across single organisms of different size and the aggregate results for entire ecosystems composed of diverse organisms, which we characterize by a distribution of cell sizes.

We first consider how to systematize stoichiometry across the diversity of microbial life using scaling laws based on cell size. We then combine these with abundance distributions to obtain a simple perspective on the bulk stoichiometry expected for a population of various cell sizes, and we demonstrate the impact that size distributions can have on these bulk stoichiometries. We then turn to a simple chemostat model of biogeochemistry where nutrients flow into an environment and interact with cellular physiology. Here, we consider the differences in cellular and fluid stoichiometry in an ecosystem composed first of a single cell size, and then of many cell sizes. This approach relies on the scaling of bulk physiological characteristics, such as nutrient quotas, with cell size, and we end by generalizing the exponents of these scaling relationships and showing the consequences this has on differences between the particulate and fluid stoichiometry. Throughout we discuss the general signatures of life that exist at either the cell and ecosystem level.

## Deriving Elemental Ratios Across Cell Size

Our interest here is in generalizing organism physiology and connecting it to stoichiometric ratio measurements that could be performed as part of astrobiological explorations of other planets using current or near-future instrumentation. Stoichiometry could be used as a relatively simple biosignature and, when considered within the context of the stoichiometry of the environment surrounding the particle/cell, could serve as a universal or agnostic biosignature. Agnostic biosignatures aim to identify patterns of living systems that may not necessarily share the same biochemical machinery as life on Earth. The need for reliable agnostic biosignatures increases as we examine planets deeper in the Solar System where common heritage with life on Earth is less likely.

Recently a variety of biological regularities have been discovered for life on Earth that show that organism physiology can be characterized by systematic trends across diverse organisms (e.g. Andersen et al. 2016; Brown et al. 2004; West and Brown 2005; Savage et al. 2004). These trends are often power-law relationships between organism size and a variety of physiological and metabolic features, and are derivable from a small set of physical and biological constraints (Kempes et al. 2019). Both physiological features and bulk organism stoichiometries have been previously shown to follow allometric scaling relationships for diverse organisms ranging from bacteria to multicellular plants (Elser et al. 1996, 2000; Vrede et al. 2004; Kerkhoff et al. 2005; Elser et al. 2010; DeLong et al. 2010; Kempes et al. 2012; Edwards et al. 2012; Kempes et al. 2016, 2017; Finkel et al. 2004, 2016a, b; Finkel 2001; Beardall et al. 2009; Tang 1995; West and Brown 2005), and so the allometric perspective taken here on stoichiometry could be applied to many levels of biological organization including entire ecosystems (e.g. Elser et al. 2000). Intuitively, these relationships can be viewed as the optimization of physiological function under fixed constraints through evolutionary processes (Kempes et al. 2019). As such, in many contexts these scaling relationships may represent universal relationships connected to fundamental physical laws such as diffusive constraints. However, in many cases the cross-species scaling may reflect emergent and interconnected constraints of the physiology itself or of evolutionary history and contingency, in which case we might expect these scaling relationships to vary across life on diverse worlds. For example, changes in the network architecture of the metabolism with size (Kim et al. 2019) could be governed by the likelihood of cross-reactivity between molecules, which could depend on what types of molecules are being employed. In general, the possibility of contingent and emergent constraints is an important consideration for astrobiology.

In microbial life, classic and recent work has systematized macromolecular abundances in terms of key properties of organisms such as overall growth rate or cell size (e.g. Shuler et al. 1979; Vrede et al. 2004; Loladze and Elser 2011; DeLong et al. 2010; Kempes et al. 2012; Edwards et al. 2012; Kempes et al. 2016, 2017; Savage et al. 2004; Tang 1995; West and Brown 2005). For example the connection between cellular growth rate and RNA and protein abundances has long been documented with various models proposing mechanisms for predicting these trends (Shuler et al. 1979; Klausmeier et al. 2004b; Vrede et al. 2004; Loladze and Elser 2011; Kempes et al. 2016). Here, we rely on work that has systematized various physiological processes and interconnections among macromolecular abundance in terms of cell size (e.g. DeLong et al. 2010; Kempes et al. 2012, 2016; Finkel et al. b; Tang 1995; West and Brown 2005), where often the interconnection between features can be systematically derived. For example, models have derived the dependence of growth rate on cell size from the cross-species scaling of metabolic rate with cell size (Kempes et al. 2012), and in turn, the ribosomal requirements given this growth rate and the scaling of protein abundance (Kempes et al. 2016). Not all of these scaling laws are understood from first principles, but they do provide a way to systematically determine macromolecular abundances from organism size. For example, bacteria follow a systematic set of scaling relationships where protein concentrations are decreasing with increasing cell size and RNA components are increasing in concentration (Kempes et al. 2016).

From the broad set of macromolecular scaling relationships it is possible to derive the elemental ratio of a cell of a given size simply by considering the abundance and elemental composition of each component. The elemental ratio of the entire ecosystem is then found by considering the size distribution of organisms.

We calculate the total elemental abundances for a cell by knowing the elemental composition of a component, $c_{i}$ (e.g. N/protein), and the total quantity of that component, $n_{i}\left( V_{c}\right)$, in a cell of a given size $V_{c}$. The total abundance of one element, *E* (*mol*/*cell*), is equal to the sum across all cellular components

1 $$\begin{aligned} E\left( V_{c}\right) =\sum _{i} c_{i}n_{i}\left( V_{c}\right) , \end{aligned}$$

where the components are major categories of macromolecules such as proteins, ribosomes, and mRNA. Each of these components has a known scaling with cell size given in Box 1. As an example, the total nitrogen content in bacteria is given by

2 $$\begin{aligned} E\left( V_{c}\right)= & {} c_{N,p}n_{protein}+c_{N,DNA}n_{DNA}+c_{N,mRNA}n_{mRNA}\nonumber \\&+c_{N,tRNA}n_{tRNA}+c_{N,ribo}n_{ribosomes}+c_{N,l}n_{l}+c_{N,e}n_{e} \end{aligned}$$

where $c_{N,p}$ is the average number of N in protein, $c_{N,DNA}$, $c_{N,mRNA}$, $c_{N,tRNA}$, and $c_{N,ribo}$ are the average N in various types of DNA and RNA, $c_{N,l}$ is the N in lipids, and $c_{N,e}$ is the N in energy storage molecules such as ATP and carbohydrates. The counts of the macromolecules are given by $n_{protein}$, $n_{ribosomes}$, $n_{DNA}$,$n_{tRNA}$, $n_{mRNA}$, $n_{l}$, and $n_{e}$ which represent the numbers of proteins, ribosomes, DNA, tRNA, mRNA, lipids, and energy storage molecules in the cell, all of which depend on cell size (Box 1). For our analysis here we focus on N:P as an illustrative case, and thus typically ignore carbohydrates and lipids, which are minor cellular sources of these elements.

**Fig. 1:**
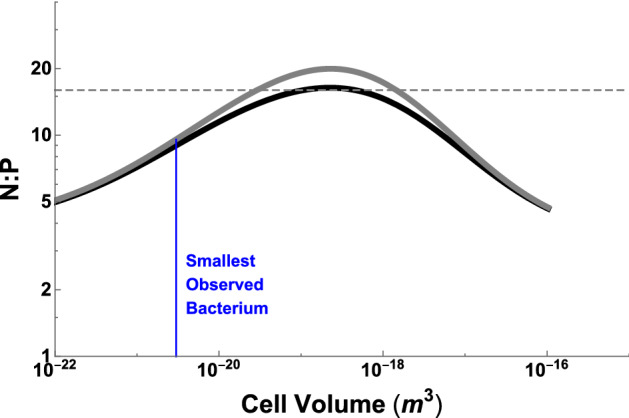
Elemental ratios as a function of bacterial cell size showing a non-constant stoichiometry that often differs from the Redfield ratio (e.g. N:P of 16:1 indicated by the dashed line) for many cell sizes. The black curve is for gram-negative bacteria and gray is for gram-positive bacteria

Using typical values for the elemental composition of each component Geider and La Roche (2002), Fig. 1 gives the ratio of elements with overall cell size. This result shows that the elemental ratios agree with Redfield for some cell sizes but deviate significantly for most bacterial cell sizes. Both small and large bacterial cells have a decreased ratio of N to P compared with the Redfield ratio. It should be noted that the Redfield ratio is known to vary widely, and do so in ways that are ecologically meaningful from a resource competition perspective (e.g. Geider and La Roche 2002; Klausmeier et al. 2004b, a, 2008). We discuss these points in greater detail below.

These observations also show that one possible agnostic biosignature is non-constant elemental ratios across particle sizes. This is the result of evolution optimizing organism physiology at different scales (Kempes et al. 2019) which will lead to different ratios of macromolecules and thus different elemental ratios at each particle size. This is true even when the set of macromolecules is largely conserved across many species but the relative ratio of these macromolecules changes due to scaling laws with cell size, as is the case with ribosomes, DNA, and proteins. The strong and consistent trend of elemental ratios with cell size should be distinctly different from the patterns of abiotic particles.

Within this overall framework it is important to consider the assumptions made by a particular model as differences in these assumptions will give rise to a variety of scaling relationships for macromolecular abundances with cell size or growth rate. For example, considering models of RNA and protein abundance, the set of past models often focuses on the tradeoffs and interconnected requirements for ribosomes and all other functional proteins (Shuler et al. 1979; Klausmeier et al. 2004b; Loladze and Elser 2011; Kempes et al. 2016). Klausmeier et al. consider the tradeoffs associated with the investment in resource acquisition or biosynthesis out of a fixed abundance of proteins in a model that couples physiology to the environment (Klausmeier et al. 2004b). This model shows that there is a different optimum for the number of ribosomes under exponential growth compared with a population that is at competitive equilibrium. Loladze and Elser consider exponential growth and define a reciprocal feedback between ribosomes and proteins, where RNA drives the rate of protein synthesis, and protein abundance drives the rate of rRNA production through RNA polymerase (Loladze and Elser 2011). This reciprocal dynamic leads to the prediction of a single homeostatic ratio of protein:rRNA, which can be calculated from biochemical parameters and where the prediction agrees with the data for several species. Kempes et al. focus on the requirement that the ribosomes replicate all proteins (including ribosomal proteins) in the time that the cell divides and takes the cellular growth rate and protein abundances (both of which systematically scale with cell size) as inputs to predict the ribosome requirement (Kempes et al. 2016). This result differs from Loladze and Elser in that it allows for a non-constant protein:rRNA ratio that depends on the distinct scaling of growth rate and protein abundance, where it is important to note that the total quantity of RNA polymerase and total quantity of all proteins could each have a distinct scaling with cell size.

For future work aimed at building general models of cell physiologies for astrobiology it is important to consider both how differences in assumptions and model complexity – which could range from the simple coupled dynamics of protein and RNA production, to whole-cell models which consider much more complicated interconnections amongst transport, metabolic, and synthesis processes (e.g. Shuler et al. 1979; Karr et al. 2012) – will lead to different predictions. For our purposes here it is sufficient to rely on models, or empirical descriptions, that match the known interspecific scaling in macromolecular abundance.

It should also be noted from a practical perspective that sampling issues may still exist. For example, it can be hard to separate biotic from abiotic particles in the Earth’s oceans using known devices (Andersson and Rudehäll 1993). However, the stoichiometry of these particles once sorted are expected to radically differ, which should be systematically verified. Addressing these issues is an important topic of future work. In addition, it is important to note that these results are based on the macromolecular abundances of cells growing at maximum rate under optimal nutrient conditions, and cells are known to respond to environmental conditions by shifting macromolecular ratios and elemental abundances (Elrifi and Turpin 1985; Healey 1985; Rhee 1978). We address these processes of acclimation in our coupled biogeochemical model.
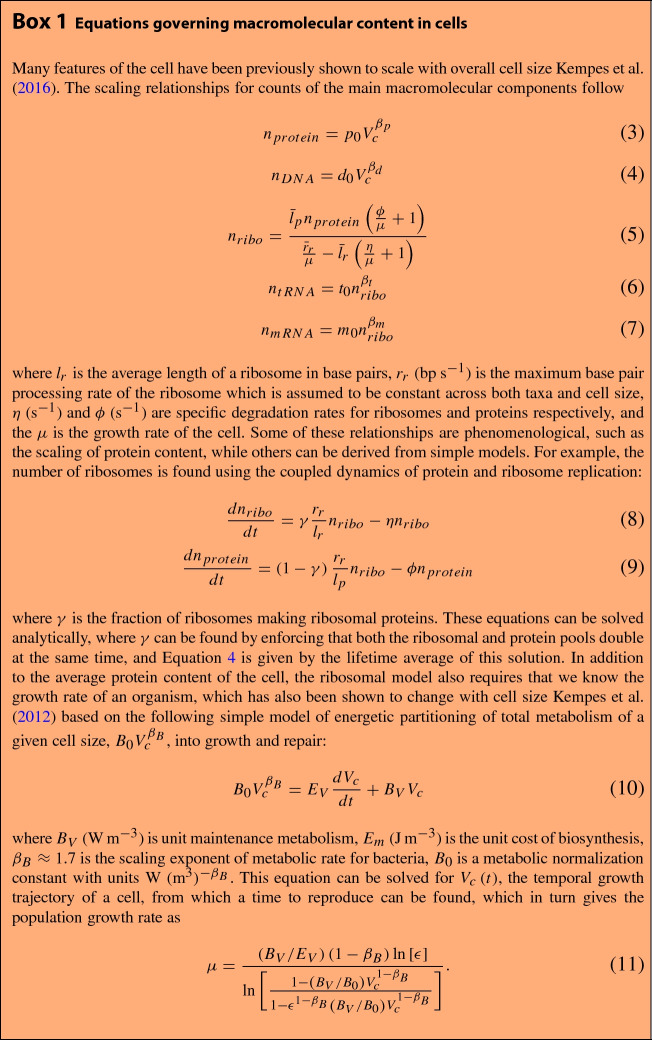

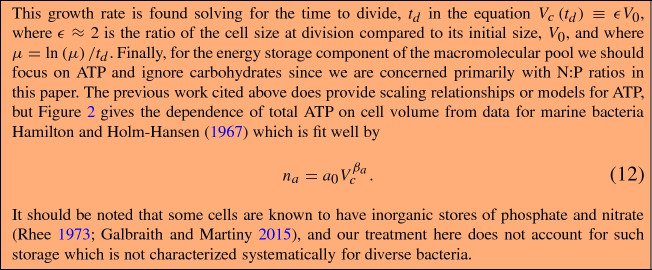

**Fig. 2:**
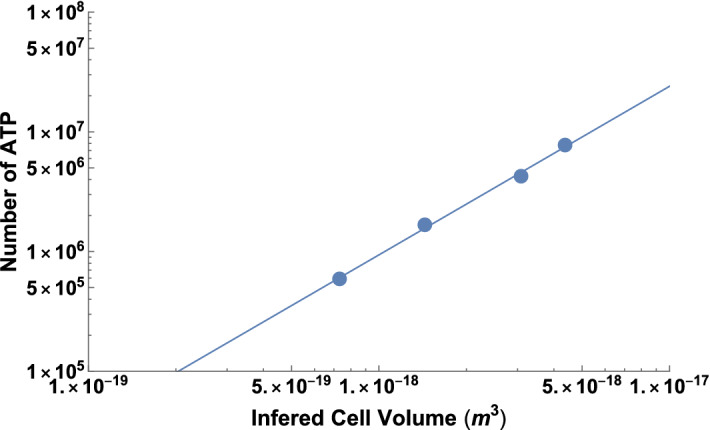
The number of ATP molecules as a function of cell volume in bacteria. The original data are from Hamilton and Holm-Hansen (1967) where the original measurement of carbon content of a cell has been converted to cell volume using the relationship in Løvdal et al. (2008), and ATP mass per cell has been converted to counts per cell. The data follow $n_{a}=a_{0}V_{c}^{\beta _{a}}$ with $\beta _{a}=1.41\pm 0.22$

## Deriving Elemental Ratios in Environments From Size Distributions

Our derivations and calculations above focus on measurements of single cells along with their size, however the most common measurement of stoichiometry, including the original Redfield measurements (Redfield 1958), is of the bulk properties of all filtered particles. Thus, it is useful to translate the above cell-level N:P ratios to whole-environment values. Here, we will consider the value found from aggregating all particulate matter, later we will address both the aggregate particulate and surrounding fluid. It is important to note that these considerations of particulate stoichiometry only account for living cells and that a complete model would need to add the contribution from abiotic particles and detritus.

Given the strong connection between cell size and elemental ratios we can determine the aggregate elemental ratio within a microbial ecosystem by simply knowing the cell-size distribution. The total concentration of one element in an environment is given by

13 $$\begin{aligned} E_{tot}=\int _{V_{min}}^{V_{max}}E\left( V_{c}\right) {\mathcal {N}}\left( V_{c}\right) dV_{c} \end{aligned}$$

where ${\mathcal {N}}\left( V_{c}\right)$ ($cells/m^{3}$ per increment of cell size) is the concentration of individuals of size $V_{c}$ in the environment (note that this equation holds for concentrations or frequencies of individuals), and $V_{min}$ and $V_{max}$ give the smallest and largest sizes, respectively.

**Fig. 3:**
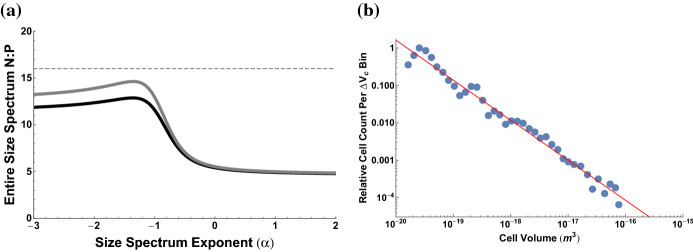
**a** Elemental ratios for an entire ecosystem given a cell size distribution characterized by ${\mathcal {F}} \propto V_{c}^{-\alpha }$ where ${\mathcal {F}}$ is frequency and $V_{c}$ is cell size. The dashed lined is the standard Redfield N:P ratio of 16:1. The black curve is for gram-negative bacteria and gray is for gram-positive bacteria. For reference, **b** gives a measured size spectrum from Cavender-Bares et al. (2001) with a fitted exponent of $\alpha =-1.07\pm 0.05$. Here the size bin is defined by $i \le \log _{10} V_{c} < i+\Delta$ with *i* taken in steps of $\Delta = 0.10$. The comprehensive data from Cavender-Bares et al. (2001) show exponents that vary between $\alpha =-0.95$ and −1.35. In **a** we used a discrete logarithmic summation to obtain total N and P concentrations.

To compare the elemental ratios we must first specify the frequency of individuals of different size. The distribution of individual sizes, often referred to as the size-spectrum, has been previously investigated in detail (e.g. Sheldon and Parsons 1967; Cavender-Bares et al. 2001; Cuesta et al. 2018; Ward et al. 2012; Taniguchi et al. 2014; Irwin et al. 2006), and is observed to follow a variety of functional forms. One commonly observed relationship is a negative power law between cell size and abundance in an environment of the form ${\mathcal {N}}\left( V_{c}\right) = C V_{c}^{-\alpha }$, where (Cavender-Bares et al. 2001) showed that exponents typically vary between $\alpha =-0.95$ and $\alpha =-1.35$ using logarithmic binning (see Fig. 3b for an example abundance relationship).

Using a logarithmically-binned discrete version of Eq. 13 with the elemental relationships $E\left( V_{c}\right)$ from the previous section, and taking ${\mathcal {N}}\left( V_{c}\right) = C V_{c}^{-\alpha }$ we can explore the range of elemental ratios as a function of $\alpha$, where the value of $\alpha$ adjusts which cell sizes are being more heavily weighted in the integral. More specifically, $\alpha =0$ weights all cell sizes equally, more negative exponents increasingly weight smaller cells, and more positive exponents increasingly weight larger cells. In Fig. 3a we have plotted the range of elemental ratios as a function of $\alpha$, where we find that only certain size distributions would produce values close to the typical Redfield ratio at the scale of an entire environment. Specifically, for $\alpha <0$, we find values that vary between 5:1 and 15:1 in the N:P ratio. The values most closely match the Redfield ratio of 16:1 for $\alpha =-1.35$ which differs slightly from the best fit exponent of $\alpha =-1.07\pm 0.05$ (Cavender-Bares et al. 2001) (Fig. 3b). However, it should be noted that characterizing the distributions of cell sizes as a power law is a simplification of more complicated distributions which often have a maximum abundance at an intermediate size (Sheldon and Parsons 1967; Cavender-Bares et al. 2001). The maximum abundance can be seen at the far left of Fig. 3b where the peaked function is well approximated by a piecewise power law with a positive exponent on the left and negative exponent on the right. If we use the exact empirical function for ${\mathcal {N}}\left( V_{c}\right)$ over the range of bacterial sizes we calculate an N:P of 12.84 for gram-negative bacteria and 14.65 for gram-positive bacteria, which closely match the Redfield ratio.

These results show that our procedure generates *a priori* expectations of whole-environment stoichiometries from particle-size distributions and known organism physiology, and could be generalized to any distribution of cell sizes and any systematic physiology (e.g. presently unknown living systems that use a set of P and N-containing biomolecules different than Earth’s proteins and nucleic acids). Even without generalizing physiology the variation in size distribution leads to a variety of total biomass N:P ratios. No single ratio can be relied on as a distinct biosignature.

## Generalized Physiological and Ecological Models of Biogeochemistry

Thus far we have seen that strong trends in N:P with particle size could be an indicator of life, but that the total stoichiometric ratio of all biomass (filtered particles) does not have a single reliable value as a biosignature because this depends on the distribution of cell sizes. This approach also considers the entire set of particulate matter in isolation without considerations of environmental conditions. We need measurements that assess the “livingness” of a particular sample in the context of its environment, and one possibility is to simultaneously measure both the particulate and environmental (fluid) stoichiometries. It is also important to consider that macromolecular and elemental abundances in cells change as cells acclimate to environmental constraints, where there are known physiological optima based on environmental conditions (Burmaster 1979; Legović and Cruzado 1997; Klausmeier et al. 2004a, b, 2007, 2008), and which is the topic of the following chemostat models for microbial life living in aquatic environments.

A variety of efforts have shown how steady-state elemental ratios can be derived from physiological models coupled to flow rates in an environment (Legović and Cruzado 1997; Klausmeier et al. 2004a, b, 2007, 2008). These chemostat models contain the simplest components of a biogeochemical model: the influx of inorganic nutrients, consumption and transformation of nutrients into cellular materials as cells grow, and the loss of both biomass and inorganic nutrients from the system. Such models are typically written as

14 $$\begin{aligned} \frac{dR_{i}}{dt}= & {} a\left( R_{i,0}-R_{i}\right) -f_{i}\left( R_{i}\right) {\mathcal {N}} \end{aligned}$$

15 $$\begin{aligned} \frac{dQ_{i}}{dt}= & {} f_{i}\left( R_{i}\right) -\mu (\vec {Q})Q_{i} \end{aligned}$$

16 $$\begin{aligned} \frac{d{\mathcal {N}}}{dt}= & {} \mu (\vec {Q}){\mathcal {N}}-m{\mathcal {N}} \end{aligned}$$

where $\mu (\vec {Q})$ is the growth rate as a function of all of the existing elemental quotas (cellular quantities), and is typically given by

17 $$\begin{aligned} \mu (\vec {Q})=\mu _{\infty }\min \left( 1-\frac{Q_{1,min}}{Q_{1}},1-\frac{Q_{2,min}}{Q_{2}},...,1-\frac{Q_{q,min}}{Q_{q}}\right) \end{aligned}$$

where *q* is the total number of limiting elements (Legović and Cruzado 1997; Klausmeier et al. 2004a, b, 2007, 2008). The function $f_{i}\left( R_{i}\right)$ is the uptake rate for a given nutrient. The terms $Q_{i}$, $\mu _{\infty }$, and $f_{i}\left( R_{i}\right)$ are all known to systematically change with cell size (see Box 2), where commonly the uptake function is given by

18 $$\begin{aligned} f_{i}=U_{max} \frac{R_{i}}{K_{i}+R_{i}} \end{aligned}$$

given the half-saturation constant $K_{i}$ and the maximum uptake rate $U_{max}$ (Burmaster 1979). In this model *a* is the flow rate of the system, which affects both the inflow of nutrients from outside the system where $R_{i,0}$ is the concentration outside the system, and the loss of the nutrients from the system. Similarly, *m* is the mortality rate of the cells and is often taken to be equal to the flow rate *a* (Klausmeier et al. 2004a, b, 2007, 2008). In this system one nutrient is typically limiting because of the minimum taken in Eq. 17, and thus the equilibria of the system are typically dictated by the exhaustion and limitation of one nutrient. Previous work has shown that growth can be maximized in this framework by considering the allocation of resources to different cellular machinery, and that this leads to two optimum physiologies, one where maximum growth rate is optimized, and another where all of the resource equilibrium values are simultaneously minimized leading to resource colimitation and neutral competitiveness with all other species (Klausmeier et al. 2004a, b).

In this model the steady-state biomass, ${\mathcal {N}}^{*}$, limiting resource, $R^{*}$, and quota of the limiting resource, $Q^{*}$, are given by

19 $$\begin{aligned} {\mathcal {N}}^{*}= & {} \frac{a\left( R_{in}-R^{*}\right) \left( \mu _{\infty }-m\right) }{Q_{min}\mu _{\infty }m} \end{aligned}$$

20 $$\begin{aligned} R^{*}= & {} \frac{Q_{min}m\mu _{\infty }K}{U_{max}\left( \mu _{\infty }-m\right) -Q_{min}\mu _{\infty }m} \end{aligned}$$

21 $$\begin{aligned} Q^{*}= & {} Q_{min}\frac{\mu _{\infty }}{\mu _{\infty }-m}, \end{aligned}$$

(Legović and Cruzado 1997; Klausmeier et al. 2004a, b, 2007, 2008) where, for extant life, the physiological features are known to depend on size according to

22 $$\begin{aligned} U_{max}= & {} U_{0}V_{c}^{\zeta } \end{aligned}$$

23 $$\begin{aligned} K= & {} K_{0}V_{c}^{\beta } \end{aligned}$$

24 $$\begin{aligned} Q_{min}= & {} Q_{0}V_{c}^{\gamma } \end{aligned}$$

25 $$\begin{aligned} \mu _{\infty }= & {} \mu _{0}V_{c}^{\eta }. \end{aligned}$$

where the empirical values for the exponents and normalization constants are provided in Box 2. Given these general physiological scaling relationships the steady states are

26 $$\begin{aligned} {\mathcal {N}}^{*}\left( V_{c}\right)= & {} \frac{a\left( R_{in}-R^{*}\right) \left( \mu _{0}V^{\eta }-m\right) }{m Q_{0}\mu _{0}V^{\gamma +\eta }} \end{aligned}$$

27 $$\begin{aligned} R^{*}\left( V_{c}\right)= & {} \frac{m Q_{0}\mu _{0}K_{0}V^{\gamma +\eta +\beta }}{U_{0}V^{\zeta }\left( \mu _{0}V^{\eta }-m\right) -mQ_{0}\mu _{0}V^{\gamma +\eta }} \end{aligned}$$

28 $$\begin{aligned} Q^{*}\left( V_{c}\right)= & {} \frac{Q_{0}\mu _{0}V^{\gamma +\eta }}{\mu _{0}V^{\eta }-m} \end{aligned}$$

where it is important to note that these equations provide results for a single cell size considered in isolation. Below we first consider how these functions change due to cell size using known physiological scaling and then general exponents, and then we derive an ecosystem-level perspective from these results and discuss potential biosignatures under a range of exponent values.
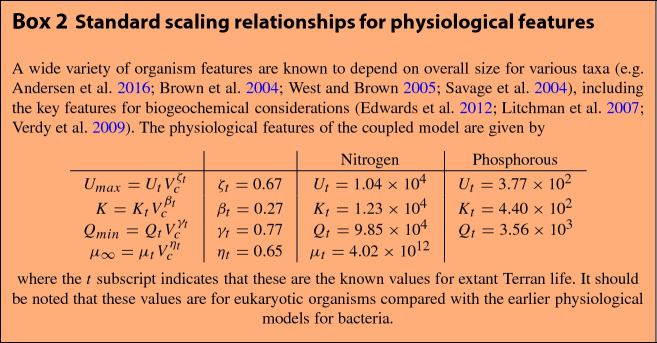


### Single-species Biogeochemistry for Extant Life

**Fig. 4:**
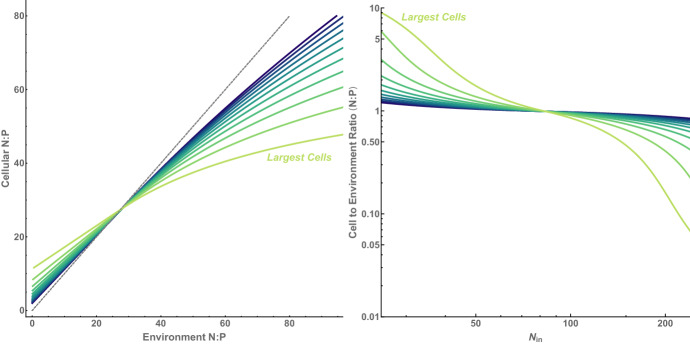
**a** Elemental ratios within cells as a function of the environmental ratio and cell size (dark blue is the smallest and light yellow is the largest cells), where the ecosystem is composed of only a single cell size. The dashed line is the one-to-one line. **b** The differences between cell stoichiometric ratios and the environment as a function of the nitrogen inflow, $N_{in}$, which is also the parameter being varied in **a**

The above model provides a simple but general biogeochemical system where cellular physiology is coupled to an environment, and can be deployed to address ecosystems of various ecological complexity. First we consider the case where an environment is dominated by a single species, which would correspond to the measurement of a consistent particle size in our framework. Taking the known physiological scaling relationships for extant life (Box 2) we find that the size of the organism has a strong effect on the stoichiometric ratios of both the particles and fluid. Figure 4 gives the steady state N:P of cells as a function of steady state environmental N:P and cell size. The variation in the steady-state environmental and cellular N:P was achieved by varying the inflow concentrations $R_{i,0}$.

We find that the largest cells will show the greatest deviation from the environmental concentration for most environmental ratios. Differences between the fluid and particle stoichiometry may define a biosignature, and these will be most noticeable for environments dominated by the largest cells. It should be noted that these results depend on the specific scaling relationships of the physiological features given in Box 2, which could greatly vary for life beyond Earth and are even known to vary across taxa for extant Terran life (DeLong et al. 2010; Kempes et al. 2012).

### Generalized ecosystem biogeochemistry

The above coupling of cells to an environment considers a biogeochemical dynamic with only a single species that is characterized by a given cell size. This is the most rudimentary possibility for an ecosystem and is generally unlikely, but considering the full range of possibilities for life in the universe, could be of relevance to particular astrobiological contexts such as environments with low energy flux and characterized by a single resource limitation. However, we would like to expand this perspective to more complicated ecosystems with greater diversity as represented by a variety of cell sizes.

Classic resource competition theory in equilibrium (e.g. Tilman 1982; Levin 1970; Hutchinson 1953, 1957; Volterra 1927, 1931) indicates that for multiple species, in our case multiple cell sizes, to coexist on a single limiting resource they must all share the same $R^{*}$ value. This is not naturally the case given the physiological scaling relationships outlined above, or the unlikelihood that many species will have identical physiological parameter values. In general, at most *x* number of species can coexist in equilibrium if there are *x* independent limiting factors (Levin 1970), and in our framework we can adjust the mortality rate, *m*, to abstractly represent the combination of many factors and to obtain coexistence. This adjustment could be the consequence of a variety of other factors such as variable predation, sinking rates, phage susceptibility, or intrinsic death. For our purposes this approach allows us to obtain a spectrum of cell sizes in connection with our earlier focus.

To enforce coexistance we take $R^{*}\left( V_{c}\right) =R_{c}$, where $R_{c}$ is a constant, in which case the required mortality rate is given by

29 $$\begin{aligned} m= & {} \frac{R_{c}U_{max}\mu _{\infty }}{R_{c}U_{max}+Q_{min}\mu _{\infty }\left( K+R_{c}\right) } \end{aligned}$$

30 $$\begin{aligned}= & {} \frac{R_{c}U_{0}\mu _{0}v^{\zeta +\eta }}{R_{c}U_{0}v^{\zeta }+Q_{0}\mu _{0}v^{\gamma +\eta }\left( R_{c}+K_{0}v^{\beta }\right) }. \end{aligned}$$

This function for *m* should be seen as the consequence of the complicated evolutionary dynamics of many species living in a coupled ecosystem where prey and predator traits have evolved over time and new effective niches have emerged. It should also be noted *m* is now size dependent compared with being set to constant value which was the case for the earlier results.

Our mortality relationship can be incorporated into ${\mathcal {N}}^{*}$ to give the scaling of biomass concentration for each cell size:

31 $$\begin{aligned} {\mathcal {N}}^{*} \left( V_{c}\right) =\frac{a\left( R_{in}-R_{c}\right) V_{c}^{-\zeta }\left( R_{c}+K_{0}v^{\beta }\right) }{R_{c}U_{0}}. \end{aligned}$$

This result has two important limits, where either the half-saturation constant is much smaller than the equilibrium value of nutrient in the environment, $K_{0}v^{\beta }\ll R_{c}$, or is much bigger than this environmental concentration, which leads to

32 $$\begin{aligned} {\mathcal {N}}^{*}\left( v\right) = {\left\{ \begin{array}{ll} \propto V_{c}^{-\zeta } &{} K_{0}V_{c}^{\beta }\ll R_{c} \\ \propto V_{c}^{\beta -\zeta } &{} K_{0}V_{c}^{\beta }\gg R_{c} \end{array}\right. } \end{aligned}$$

These two relationships provide nice bounds on the scaling of ${\mathcal {N}}$ given the underlying physiological dependencies.

Similarly, the quota is given by

33 $$\begin{aligned} Q^{*}=Q_{0}V_{c}^{\gamma }+\frac{R_{c}v^{\zeta -\eta }U_{0}}{\mu _{0}\left( R_{c}+K_{0}v^{\beta }\right) }. \end{aligned}$$

which implies that the ratio of particle to fluid elemental abundance for the limiting nutrient is the following function of cell size

34 $$\begin{aligned} \frac{{\mathcal {N}}^{*}Q^{*}}{R^{*}}= {\left\{ \begin{array}{ll} \frac{a (R_{in}-R_{c}) \left( Q_{0} V_{c}^{\gamma -\zeta }+\frac{U_{0} V_{c}^{-\eta }}{\mu _{0}}\right) }{R_{c} U_{0}} &{} K_{0}V_{c}^{\beta }\ll R_{c} \\ \frac{a (R_{in}-R_{c}) \left( K_{0} Q_{0} \mu _{0} V_{c}^{\beta +\gamma -\zeta }+R_{c} U_{0} V_{c}^{-\eta }\right) }{ R_{c}^2 U_{0}\mu _{0}} &{} K_{0}V_{c}^{\beta }\gg R_{c} \end{array}\right. } \end{aligned}$$

This relationship is similar to the types of results shown in Fig. 4, but gives the ratio between cell and environment concentrations for a single element of interest (rather than as comparisons of ratios of elements), and importantly, does so under the constraints of coexistence. This result leads to particular biosignature possibilities when measuring only a single element, and does so for the more realistic ecosystem conditions of coexistence. If we measure the particle size distribution in an environment, then this is enough to specify the value of $\alpha =-\zeta$ or $\alpha =\beta -\zeta$ from Eq. 34, leaving us with $\gamma$ and $\eta$ to determine the element ratio scaling between cells and the environment as a function of particle size.

From a biosignatures perspective, the most ambiguous measurement would be particles that perfectly mirror the environmental stoichiometry where $N^{*}Q^{*}/R^{*}$ equals a constant for all particle sizes. In the first limit, $K_{0}V_{c}^{\beta }\ll R_{c}$, this would require $\zeta =\gamma =-\alpha$ and $\eta =0$. This result would imply that the quota and uptake rates would need to scale with the same exponent and as the negative value of the size exponent, both of which are consistent with the observations of Box 2 and Fig. 3b for extant life. However, this result also requires that there would be no change in growth rate with cell size, which is very unlikely from a variety of biophysical arguments.

In the second limit, $K_{0}V_{c}^{\beta }\gg R_{c}$, a constant value of $N^{*}Q^{*}/R^{*}$ requires that $\zeta -\beta =\gamma =-\alpha$ and $\eta =0$. Again the absence of changes in growth rate connected with $\eta =0$ is unlikely. In addition, under this scenario the difference in the uptake and half-saturation scaling, represented by $\zeta -\beta$, must equal the scaling of the quota and take the opposite value as the size-spectrum scaling, which is a combination that is not consistent with extant life and is a very special case in general. Thus, under both limits $N^{*}Q^{*}/R^{*}$ is unlikely to have a constant value as a function of cell size, and an observed scaling in this ratio forms a likely biosignature.

This potential biosignature still requires one to measure the cell-size spectrum in detail, which may be challenging in certain settings or with certain devices. However, these relationships can be easily translated into an aggregate ecosystem-level measurement by averaging over all coexisting cells, where the average is given by

35 $$\begin{aligned} \left\langle \frac{\mathcal {N}^{*} Q^{*}}{R^{*}}\right\rangle= & {} \frac{1}{V_{max}-V_{min}}\int _{V_{min}}^{V_{max}}\frac{\mathcal {N}^{*}\left( V\right) Q^{*}\left( V\right) }{R^{*}}dV \end{aligned}$$

36which, considering the two approximations for ${\mathcal {N}}$, becomes37To fully specify this community level ratio for generalized life we need to constrain the normalizations constants, $Q_{0}$, $k_{0}$, $U_{0}$, and $\mu _{0}$ given any choice of the exponents. A reasonable way to determine the values of these constants is to match the generalized rates to the observed Terran rates from Box 2 at a particular reference size, $V_{r}$, which leads to

38 $$\begin{aligned} U_{0}= & {} U_{t}V_{r}^{\zeta _{t}-\zeta } \end{aligned}$$

39 $$\begin{aligned} K_{0}= & {} K_{t}V_{r}^{\beta _{t}-\beta } \end{aligned}$$

40 $$\begin{aligned} Q_{0}= & {} Q_{t}V_{r}^{\gamma _{t}-\gamma } \end{aligned}$$

41 $$\begin{aligned} \mu _{0}= & {} \mu _{t}V_{r}^{\eta _{t}-\eta }. \end{aligned}$$

**Fig. 5:**
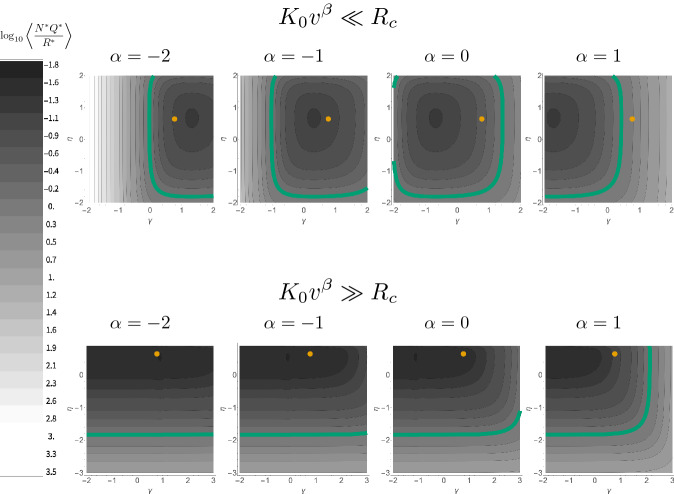
The ratio of the cellular to environmental nitrogen, $\left\langle {\mathcal {N}}^{*} Q^{*}/R^{*}\right\rangle$, as function of the size spectrum exponent $\alpha$, the minimum quota (cellular requirement) scaling exponent, $\gamma$, and the growth rate scaling exponent, $\eta$. We have shown the results for the two approximations $K_{0}v^{\beta }\ll R_{c}$ and $K_{0}v^{\beta }\gg R_{c}$. In each plot the green line represents $\left\langle {\mathcal {N}}^{*} Q^{*}/R^{*}\right\rangle =1$, or $\log _{10}\left\langle {\mathcal {N}}^{*} Q^{*}/R^{*}\right\rangle =0$. The orange point represents the known $\gamma$ and $\eta$ exponent values for extant life from Box 2

After calibrating the constants to an intermediate cell size of $V_{r}=10^{-18}$ ($\hbox {m}^{3}$), Fig. 5 gives the community level $\left\langle {\mathcal {N}}^{*} Q^{*}/R^{*}\right\rangle$ as a function of the scaling exponents. When $K_{0}v^{\beta }\ll R_{c}$ the size exponent $\alpha$ specifies $-\zeta$, and when $K_{0}v^{\beta }\gg R_{c}$ then $\alpha$ specifies $\beta -\zeta$. In both approximations we plot $\left\langle {\mathcal {N}}^{*} Q^{*}/R^{*}\right\rangle$ as a function of $\eta$ and $\gamma$ for a range of $\alpha$ values.

We find that typically $\left\langle {\mathcal {N}}^{*} Q^{*}/R^{*}\right\rangle$ differs from 1 for a wide range of $\alpha$, $\eta$ and $\gamma$ values. This is true under both limits. For fixed values of $\alpha$ the $\left\langle {\mathcal {N}}^{*} Q^{*}/R^{*}\right\rangle =1$ line is a closed curve as a function of $\eta$ and $\gamma$ (Fig. 5). This curve defines the regime within which it is possible to find $\left\langle {\mathcal {N}}^{*} Q^{*}/R^{*}\right\rangle =1$ for any value of either $\eta$ and $\gamma$, and this region covers a wide range of exponent values. However, the known values of $\eta$ and $\gamma$ for extant life occur fairly far from this curve and would show elemental concentrations that are distinguishable from the environment. It is likely that the full range of $\alpha$, $\eta$ and $\gamma$ combinations explored here are precluded for biophysical reasons, but this requires more detailed work in the future. Finally, it is important to note that most $\alpha$, $\eta$ and $\gamma$ combinations would yield cell-to-environment ratios that significantly differ from 1, and that the gradients are very steep around the $\left\langle {\mathcal {N}}^{*} Q^{*}/R^{*}\right\rangle$=1 line. Thus, it is a fairly safe assumption that the elemental abundances of cells should differ from the environment as this would be the expectation for physiological scaling chosen at random.
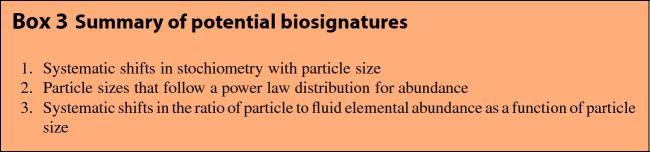


## Discussion

The general framework provided here should make it possible to assess biosignatures for a wide diversity of potential life (see Box 3 for a summary). We focused on bacterial life as an example of what we would expect in ecosystems dominated by the simplest life. However, all of our results could be tuned to other classes of organisms with the appropriate changes in scaling relationships for macromolecular content and abundance distributions. Our results do this generally for any life that is governed by a set of physiological scaling relationships, where, for example, the nutrient quotas are abstracting the underlying changes in macromolecules and could represent a diverse set of alternate physiologies and sets of macromolecules for alternate evolutionary histories or origins of life. Even for extant life on Earth the typical stoichiometry varies significantly within and across taxa (Geider and La Roche 2002; Klausmeier et al. 2004b, a, 2008; Loladze and Elser 2011; Neveu et al. 2016; Sterner et al. 2008; Vrede et al. 2004; Elser et al. 2000; Liefer et al. 2019; Finkel et al. 2016a, b), for example, plant leaves have an N:P of 30 rather than 16 (Elser et al. 2010; Kerkhoff et al. 2005). However, the main assumption in our generalized physiological model is that life will fall along allometric scaling relationships, which occurs for multiple taxa on Earth and has good justification from various arguments connected with universal physical constraints.

It should also be noted that many of the physiological scaling relationships have strong physical principles motivating the exponents and the wide variation taken in the generalized equations may not be realizable by life anywhere in the universe. Thus observed biosignatures may be much more similar to our analyses in Figs. 1, 3, and 4 than the possibilities encapsulated in our generalized physiological model as capture in Figure 5.

In addition, our efforts here have often focused on the assumption of one limiting nutrient. However, this scenario of a single resource typically does not lead to coexistence (e.g. Tilman 1982; Levin 1970; Hutchinson 1953, 1957; Volterra 1927, 1931). The problem of coexistence can be solved by many additional considerations such as environmental stochasticity, the addition of spatial dynamics, or species adaptation (e.g. Hutchinson 1953; Levins and Culver 1971; Klausmeier and Tilman 2002; Kremer and Klausmeier 2013), all of which could be important for future modeling efforts or for measurements of the spatial variation in stoichometry. However, our solution for mortality allows for coexistence in a single environment and our model is compatible with measurements made at a single or coarse-grained location which may be typical of many future astrobiological measurements. Our general physiological perspective should be combined with more advanced biogeochemical models that consider many nutrients, including trace elements, and more complex ecological and evolutionary dynamics — many of which can be connected systematically with size (Andersen et al. 2016; Kempes et al. 2019) — to fully explore the range of particle size distributions, and the particle to fluid stoichiometric differences that can be reasonably expected to represent biosignatures. Finally, since our approach only considers the living component of particulate matter future models should incorporate the stoichiometric contributions of abiotic particles and detritus along with more complex geochemistry and ask how much this addition can shift the general biosignatures presented here.

## Appendix: Macromolecular Constants for Stoichiometry

One of our overall goals is to assess the size-dependence of elemental ratios. To do this we relied on average elemental abundances of various macromolecules coupled with the number scaling of those macromolecules with cell size. Here, we provide more details on the conversion of macromolecular quantities and some novel quantification of the scaling of macromolecular abundance. For all of the elemental abundances of N and P in particular macromolecules we rely on Geider and La Roche (2002) unless otherwise indicated. Most of the scaling relationships follow from Box 1 converted into molecule numbers. It should be noted that several additional features could shift the following calculations, such as gram-positive vs. gram-negative architectures, or the observation of scaling relationships between the total membrane-bound proteins and cell size. We have included a gram-negative and gram-positive comparison in Fig. 1 to provide a sense of the range of values that these variations can cause.

*Amino Acids* We used the scaling of total protein mass for bacteria (Kempes et al. 2016) combined with the average mass of an individual amino acid, $1.79\times 10^{-22}$ g (Bremer et al. 1996), to obtain the total number of amino acids in the cell.

*Nucleotides* We used the total ribosome number count from Kempes et al. (2016) combined with the number of RNA nucleotides in a ribosome, 4566 (Bremer et al. 1996), to obtain the total number of RNA molecules contributed from ribosomes. For the mRNA we used the number of mRNA per ribosome, 1.08 (mRNA/ribosome) (Kempes et al. 2016), the average length an mRNA, 975 nucleotides (see Kempes et al. 2016 for a review), and the number of ribosomes to estimate the total abundance. Similarly, for the tRNA we used the tRNA per ribosome of 9.3 (Bremer et al. 1996), and the average length of a tRNA, 80 nucleotides (Bremer et al. 1996). For DNA we used the base pair count from Kempes et al. (2016).

*Phospholipids and Peptidoglycan* For the phospholipids we consider that $p=0.30$ of outer membrane is composed of proteins (Szalontai et al. 2000). For the remaining surface area we consider that a single phospholipid occupies $s=55\times 10^{-20}$ $\hbox {m}^{2}$ (Nichols and Deamer 1980) of surface area, such that for a gram-negative bacterium the total number of phospholipids is given by $8 \pi (1-p) \left( r_{c}^{2}+(r_{c}-\delta )^{2}\right) /s$, where $r_{c}$ is the radius of the cell and $\delta =3.43\times 10^{-8}$ m is the distance between the plasma and outer membranes (Meroueh et al. 2006). For a gram positive bacterium the total number of phospholipids can be approximated by $4 \pi (1-p) \left( r_{c}-t_{p}\right) ^{2}/s$, where $t_{p}=6.4\times 10^{-9}$ m is the thickness of the peptidoglycan layer (Meroueh et al. 2006).

For the peptidoglycan layer we take the basic monomer of NAG-NAM and associated peptides from Meroueh et al. (2006), which contains 8 N and no P and is arranged into a unit volume defined by 6 strands each of which is 8 monomers long such that the unit volume contains 384 N. This unit volume is an approximate cylinder with a diameter of $\approx 7\times 10^{-9}$ m and a height of $\approx 1\times 10^{-8}$ m (Meroueh et al. 2006). The total volume of peptidoglycan in the cell is then given by $4/3\pi \left( (r_{c}-t_{0}-t_{peri})^{3}-(r_{c}-t_{o}-t_{peri}-t_{p})^{3}\right)$ for a gram-negative bacterium, where $t_{o}=6.9\times 10^{-9}$ m is the thickness of the outer membrane and $t_{peri}=2.1\times 10^{-8}$ m is the thickness of the periplasm (Meroueh et al. 2006). For a gram-positive bacterium the total peptidoglycan volume is approximately $4/3\pi \left( r_{c}^{3}-(r_{c}-t_{p})^{3}\right)$. Both volumes allow us to count the number of unit volumes and associated elemental abundances.

*ATP* For the ATP we use the scaling from Fig. 2.
